# Comparison of two simulators for individual based models in HIV epidemiology in a population with HSV 2 in Yaoundé (Cameroon)

**DOI:** 10.1038/s41598-021-94289-z

**Published:** 2021-07-19

**Authors:** Diana M. Hendrickx, João Dinis Sousa, Pieter J. K. Libin, Wim Delva, Jori Liesenborgs, Niel Hens, Viktor Müller, Anne-Mieke Vandamme

**Affiliations:** 1 I-BioStat, Data Science Institute, Hasselt University, Hasselt, Belgium; 2 Department of Microbiology, Immunology and Transplantation, Rega Institute for Medical Research, Clinical and Epidemiological Virology, KU Leuven, 3000 Leuven, Belgium; 3 Global Health and Tropical Medicine (GHTM), Unidade de Microbiologia Médica, Instituto de Higiene e Medicina Tropical, Universidade Nova de Lisboa, Lisbon, Portugal; 4 Artificial Intelligence Lab, Department of Computer Science, Vrije Universiteit Brussel, 1050 Brussels, Belgium; 5 The South African Department of Science and Technology-National Research Foundation (DST-NRF) Centre of Excellence in Epidemiological Modelling and Analysis (SACEMA), Stellenbosch University, Stellenbosch, South Africa; 6 Department of Global Health, Faculty of Medicine and Health, Stellenbosch University, Stellenbosch, South Africa; 7 International Centre for Reproductive Health, Ghent University, Ghent, Belgium; 8 School for Data Science and Computational Thinking, Stellenbosch University, Stellenbosch, South Africa; 9 Expertise Centre for Digital Media, Hasselt University – tUL, Diepenbeek, Belgium; 10 Centre for Health Economics Research and Modelling Infectious Diseases, Vaccine & Infectious Disease Institute, University of Antwerp, Antwerp, Belgium; 11 Institute of Biology, Eötvös Loránd University, Budapest, Hungary; 12 Department of Microbiology, Immunology and Transplantation, Institute for the Future, Rega Institute for Medical Research, KU Leuven, 3000 Leuven, Belgium

**Keywords:** Infectious diseases, HIV infections

## Abstract

Model comparisons have been widely used to guide intervention strategies to control infectious diseases. Agreement between different models is crucial for providing robust evidence for policy-makers because differences in model properties can influence their predictions. In this study, we compared models implemented by two individual-based model simulators for HIV epidemiology in a heterosexual population with Herpes simplex virus type-2 (HSV-2). For each model simulator, we constructed four models, starting from a simplified basic model and stepwise including more model complexity. For the resulting eight models, the predictions of the impact of behavioural interventions on the HIV epidemic in Yaoundé-Cameroon were compared. The results show that differences in model assumptions and model complexity can influence the size of the predicted impact of the intervention, as well as the predicted qualitative behaviour of the HIV epidemic after the intervention. These differences in predictions of an intervention were also observed for two models that agreed in their predictions of the HIV epidemic in the absence of that intervention. Without additional data, it is impossible to determine which of these two models is the most reliable. These findings highlight the importance of making more data available for the calibration and validation of epidemiological models.

## Introduction

Mathematical modelling has been widely applied to better understand the transmission, treatment and prevention of infectious diseases. The role of mathematical models in understanding the dynamics of the human immunodeficiency virus (HIV) epidemic has been recently reviewed by Geffen and Welte^1^. They present examples of HIV models that were used for estimating the size of the epidemic in specific subpopulations such as the Black population in South Africa and for estimating the impact of interventions such as the use of antiretrovirals and condoms to reduce HIV and AIDS.

When considering mathematical models, two commonly used types of implementation can be distinguished: compartmental and individual-based models (IBMs). While compartmental models simulate population counts, IBMs (also called agent-based models or micro-simulation models) keep track of the history of each individual in the population separately.

Recent applications of compartmental HIV models include testing the effect of different assumptions for HIV dynamics on the predicted impact of antiretroviral therapy (ART) in men-having-sex-with-men (MSM)^2^, and the study of the influence of concurrent partnerships on HIV dynamics^3^. IBMs have been recently applied to assess the influence of pre-exposure prophylaxis (PrEP) in MSM^4^, to evaluate the long-term effect of early ART initiation^5^ and to understand the factors underlying the emergence of HIV in humans^6^.

In contrast to compartmental models, IBMs allow to model heterogeneous interactions between individuals^7^ (e.g. regarding (sexual) risk behaviour, age demography and individual response to treatment). This is an advantage when heterogeneity matters for the particular question/process that is studied. Because individual heterogeneity is inherent in transmission, prevention and treatment of HIV and other sexually transmitted infections (STI), IBMs are particularly suitable to estimate the most beneficial intervention for specific individuals. Furthermore, IBMs allow for explicit modelling of the sexual relationships that jointly form the sexual network over which STI/HIV infections are transmitted^8^.

Model comparisons are crucial for providing robust evidence for decision-making in public health and policy purposes^9^. To assess uncertainty, it is necessary to study how differences in model properties influence their prediction. Eaton et al. compared ten mathematical models aimed at studying HIV prevalence, incidence and ART^10^. However, in contrast to the models in our study, eight of these models were compartmental, and the majority of these models did not include the co-factor effects of other STIs. The remaining two models were discrete-time IBMs.

The inclusion of an STI is important in HIV modeling, as previous studies have shown that individuals infected with HIV have a higher risk for acquiring an STI^11^ and vice versa^12^. Furthermore, the risk for transmitting HIV to another person is higher in HIV-STI co-infected people than in HIV mono-infected individuals^12^. In sub-Saharan African cities, including Yaoundé (Cameroon), HSV-2 infection has been identified as an important factor influencing the spread of HIV^13^.

This study presents and compares eight models generated with two IBM frameworks for simulating HIV transmission dynamics with STI co-factor effects. The purpose of the comparison was to assess how differences in properties between the eight models influence the prediction of the impact of behavioural interventions on the HIV epidemic. For this purpose, we chose the HIV epidemic in the heterosexual population in Yaoundé (Cameroon) during the period 1980–2005, and calibrated all models to HIV prevalence time series from Yaoundé.

## Methods

### Individual-based models

In this study, individual-based models developed with the Simpact Cyan 1.0 and StepSyn 1.0 modelling frameworks, were compared. We used these two HIV model simulators for comparison because they can both be used with the popular language R^14^, and both implement dynamic sexual networks and an STI co-factor effect. Simpact Cyan 1.0 models all stages of HIV (acute, chronic, AIDS stage, final AIDS stage), while StepSyn 1.0 does not distinguish between AIDS stage and final AIDS stage (see Sects. 1.3. and 1.4. of the Supplementary Material). Only StepSyn 1.0. models the natural history of HSV-2, including ulcerative recurrences. In Simpact Cyan 1.0, no distinction between HSV-2 stages is made. A short description of each modelling framework is given below. More details about differences between the two modelling frameworks and the generation of the sexual networks are provided in Table 1 and the Supplementary Material, Sect. 1. For each modelling framework, four models were generated, starting from a simplified basic model and incorporating additional complexity. A population of 9184 individuals was simulated, of which 5000 were males and 4184 were females, corresponding with the adult sex ratio (M:F) in Yaoundé in 1997 (see “Data used for fitting Simpact Cyan 1.0 and StepSyn 1.0 models” section). Models were run without intervention and with behavioural interventions aimed at reducing the number of partners. Male circumcision, condom use and HIV antiretroviral therapy (ART) were not modeled in this study, as these interventions were not included in one or both of the modeling frameworks.

**Table 1:** Comparison of properties of models developed with Simpact Cyan 1.0 and StepSyn 1.0.

Model property	Simpact Cyan 1.0	StepSyn 1.0
Simulation framework	Individual-based model	Individual-based model
Implementation language	C + + with R and Python interfaces	R
Source code freely available	Yes	After the publication of the manuscript describing the modelling framework in detail
Update of the state of the model system	Event-driven: the state of the model system is updated each time an event happens	Fixed time steps of one week, with the creation and dissolution of sexual relationships and STI and HIV transmission
Description of stochastic processes	By hazard functions	By probability distribution functions called in each time step
Age-structured	Yes	No
Entering the population	Birth (birth event)	Sexual debut (birth-rate), immigration
Leaving the population	AIDS- and non-AIDS mortality (mortality event, age-dependent)	AIDS- and non-AIDS mortality (mortality rate), emigration
Formation and break-up of relationships within the sexual network	Formation and dissolution event. Timing of events sampled from a probability distribution resulting from carefully chosen hazard functions	Each time step, using an individual-specific probability of forming a new relationship
Individual variation in sexual behaviour	In the formation hazard, the eagerness of a person to form a relationship and the preferred age gap are used, to allow for individual variation in sexual behaviour. Both values are drawn from user-specified probability distributions	Number of short term relationships follows a power-law distribution
HIV natural history/stages	Yes (acute phase, chronic phase, AIDS stage)	Yes (acute phase, chronic phase, AIDS stage)
Other STIs as co-factor for HIV infection	Yes (implemented as a simplified STI transmission hazard)	Yes (syphilis, chancroid, gonorrhoea, chlamydia, HSV-2)
Natural history/stages—other STIs	No, stages are not explicitly modelled	Yes, with genital ulcers and discharge explicitly modelled
Pregnancy	Yes, as the period between a conception event and a birth event	No
Diagnosis/treatment of HIV	Yes	No
HIV seeding	A fraction to specify the probability of each person in the group to be a seeder or an amount to specify the number of seeders. Fraction or amount is taken from the number of people in the population	Either a random binomial for HIV seeding (seeding varies between simulations) or a fixed fraction to specify the fraction of seeders; this fraction is taken from the number of males and the number of females separately
Interventions	Behavioural interventions, HIV treatment	Behavioural interventions, circumcision, treatment of syphilis, chancroid, gonorrhoea, chlamydia, HSV-2
individual variation of viral load	Implemented in HIV transmission hazard	No
Computational time for 10,000 simulations*	~ 35 h	~ 5 h

*On 100 cores of the VSC (Xeon E5-2680v2 CPUs 2.8 GHz, 25 MB level 3 cache). Population size ~ 9000; time frame of the simulation = 35 years; parameters: see Table S10 (Simpact Cyan 1.0 basic model) and S14 (StepSyn 1.0 basic model).

#### Simpact Cyan 1.0 modelling framework

Simpact Cyan 1.0 (http://www.simpact.org/)^15^ is a freely available framework for developing IBMs to simulate HIV transmission, progression and treatment. Models developed with Simpact Cyan 1.0 are event-driven, which means that the models are not updated at fixed time intervals, but at every time an event happens, making Simpact Cyan 1.0 a continuous-time simulation modelling framework. Events occur as a result of stochastic event processes, described by hazard functions. The timing of an event is determined using the modified next reaction method (mNRM)^16^.

To initialize a model, a number of individuals are generated. The age of each person is drawn from an age distribution based on a population pyramid, and when the age is larger or equal to the sexual debut age, a person is marked as sexually active.

In Simpact Cyan 1.0, the user can specify which events are possible in the simulation, depending on the research question he or she wants to answer. The possible events are heterosexual and homosexual relationship formation and dissolution, conception and birth, HIV transmission, AIDS and non-AIDS mortality, HIV diagnosis and treatment, HIV treatment dropout, and STI transmission events (in this study HSV-2). Furthermore, events describing the natural history of HIV are implemented in Simpact Cyan 1.0. In the present study, homosexual relationships and HIV treatment are not included. For each event, the form and the parameters of the hazard function can be modified flexibly. The HIV transmission hazard can be described in terms of an individual’s viral load. In the basic model in this study, this option, as well as birth and age-dependent non-AIDS mortality, is turned off. Simpact Cyan 1.0 is implemented in C++ with R^14^ and Python^17^ interfaces.

In Simpact Cyan 1.0, HIV transmission occurs through a HIV transmission event, which is scheduled when a HIV infected person forms a relationship with another individual.

#### StepSyn 1.0 modelling framework

StepSyn 1.0 is an IBM modelling framework that simulates epidemics of STIs, including HIV. It focuses on the epidemiological synergy and interactions between HIV and other STIs. Time is divided into fixed one-week intervals. Formation and dissolution of sexual relationships and STI and HIV transmission are modelled as stochastic processes. Occurrence of HIV transmission is described by the transmission probability per sex act. For each STI, life history is explicitly modelled, including several stages, symptomatic recurrences and genital ulcers. The effects of these symptoms on the HIV transmission probability are explicitly modelled. The parameters for the timing and duration of the STI stages and symptoms and the co-factor effects of genital ulcers on HIV transmission are based on a literature review. In the present study, StepSyn 1.0 is parameterized to track only HIV and Herpes simplex virus type 2 (HSV-2). In the basic model the latter virus is modelled without explicit stages or symptomatic recurrences, and with the probability of transmission and co-factor effect on HIV both constant and not dependent on recurrences. In this study, StepSyn is ran in both this basic model, and in the full model in which HSV-2 stages and recurrences are modelled. Heterosexual relationships can include marital and short-term links and contacts between commercial sex workers (CSW) and clients. In the present study, CSWs are not included. Individuals vary in their tendency to form short-term relationships so that their number of non-marital partners within a year would follow a power-law distribution. The parameters of the latter were derived from behavioural data gathered in the 4 Cities Study^18,19^. StepSyn is implemented in R^14^. StepSyn is not yet freely available but will become so upon publication of a manuscript that describes the modelling framework into more detail (manuscript in preparation).

#### Models used in this study

The following models were used in this study:

1. Simpact Cyan 1.0 basic model (Si_Ba). This simplified model assumes no population inflow and no outflow by other causes than AIDS deaths. Furthermore, HIV transmission does not depend on an individual’s viral load. HSV-2 is implemented as a generic STI co-factor, without modelling the natural history. This means that only the impact of HSV-2 on HIV acquisition and transmission, and vice versa, are implemented.
2. Simpact Cyan 1.0 model with population inflow and outflow (Si_IO). Birth is implemented as an event related to relationship formation, followed by a conception event, using the default settings of Simpact Cyan 1.0. Non-AIDS mortality is age-dependent and is derived from the 1980 population pyramid of Yaoundé^20^. Other settings are the same as in the Si_Ba model.
3. Simpact Cyan 1.0 model implementing viral load (Si_VL). HIV transmission depends on an individual’s viral load. Other settings are the same as in the Si_Ba model.
4. Simpact Cyan 1.0 model implementing population inflow, outflow and viral load (Si_IO_VL). Birth and non-AIDS mortality are implemented in the same way as for the Si_IO model. HIV transmission depends on an individual’s viral load.
5. StepSyn 1.0 basic model (St_Ba). This simplified model assumes no population inflow (birth, immigration) and no outflow by other causes than AIDS deaths. Furthermore, HSV-2 is modelled without explicit stages or recurrences, and with the probability of transmission and co-factor effect on HIV both constant and not dependent on recurrences.
6. StepSyn 1.0 model with population inflow and outflow (St_IO). This model implements birth, non-AIDS mortality, immigration and emigration. All of these variables are expressed as a rate. Other settings are the same as in the St_Ba model.
7. StepSyn 1.0 model with the full set of HSV-2 co-factor assumptions (St_RG). HSV-2 life history is explicitly modelled, including several stages, recurrences and genital ulcers. The effects of these symptoms on the HIV transmission probability are explicitly modelled. Other settings are the same as in the St_Ba model.
8. StepSyn 1.0 model with population inflow, outflow and the full set of HSV-2 co-factor assumptions (St_IO_RG). This model implements birth, immigration, non-AIDS mortality and emigration in the same way as the Si_IO model. For HSV-2, the same settings as in the St_RG model are used.

Table 2 summarizes the properties of the 8 models mentioned above.

**Table 2:** Overview of the properties of the 8 models used in this study.

Model abbreviation	Population inflow and outflow	Birth	Non-AIDS mortality	Immigration	Emigration	Viral load	HSV-2 co-factor effect	HSV-2 stages/recurrences
Si_Ba							✓	
Si_IO	✓	✓	✓				✓	
Si_VL						✓	✓	
Si_IO_VL	✓	✓	✓			✓	✓	
St_Ba							✓	
St_IO	✓	✓	✓	✓	✓		✓	
St_RG							✓	✓
St_IO_RG	✓	✓	✓	✓	✓		✓	✓

Checkmarks indicate the features that are included in the models.

### Data used for fitting Simpact Cyan 1.0 and StepSyn 1.0 models

For the comparison of the two models, demographic data for 1997, and HIV prevalence data for 1989–1998 related to the African city of Yaoundé (Cameroon), are used. The adult male population (15–59 years) for that year was estimated at 387,398, in the 4 Cities Study^21^. Based on the surveys made by the same study (age range 15–49 years), 34.5% of men and 44.2% of women were married; and 7.2% of married men were polygamous^19^. Assuming most of the latter had 2 wives, we estimate the number of women as 387,398 × 0.345 × (1 + 0.072)/0.442 = 324,152, implying an adult sex ratio (M:F) of 1000:836.74.

Since one of our aims here is to compare HIV prevalence curves resulting from fitting HIV transmission parameters of our two models to data, we gathered HIV-1 prevalence data for Yaoundé for the period 1989–1998. The only prevalence data available were of pregnant women, for whom prevalence increased from 0.7% in 1989 to 5.5% in 1998 (US Census Bureau, 2001, HIV/AIDS Profile, Cameroon, HIV/AIDS Surveillance Database^22^) as shown in Table 3 (2nd column).

**Table 3:** HIV-1 prevalence data for Yaoundé’s pregnant women.

Year	HIV-1 prevalence (%) pregnant women	Estimated HIV-1 prevalence (%) men	Estimated HIV-1 prevalence (%) women
1989	0.71	0.609	1.159
1990	1.32	1.133	2.154
1991	2.10	1.802	3.427
1992	1.91	1.639	3.117
1993	1.30	1.115	2.122
1994	3.00	2.574	4.896
1995	2.72	2.334	4.439
1996	4.81	4.127	7.850
1997	Not available	4.100	7.800
1998	5.51	4.728	8.992

*Source* US Census Bureau, 2001, HIV/AIDS Profile, Cameroon, HIV/AIDS Surveillance Database^22^ (confidence interval and sample size not available) (second column). Third and fourth column: Estimated HIV-1 prevalences for Yaoundé’s men and women, that were used as model calibration targets in this study.

Because StepSyn 1.0 does not implement pregnant women as a separate category of the population, and we wanted to compare model simulations for males and females separately, the prevalences for pregnant women were converted to prevalences for males and females as follows. In 1997, during the 4 Cities Study, HIV-1 prevalence was 4.1% (95% confidence interval: CI 3.0–5.7%; sample size: n = 896) in men (age 15–49 years) and 7.8% (CI 6.2–9.6%; n = 1017) in women (age 15–49 years)^23^. The prevalence for pregnant women in 1997 was estimated by fitting a smoothing spline through the data from Table 3 (2nd column), using the smooth.spline function of the stats package in R, and evaluating the spline at the time point corresponding with 1997 (see Fig. S1). We obtained a prevalence of 4.778% for pregnant women in 1997 and calculated the prevalence ratio of men/pregnant women (resp. women/pregnant women) by dividing 4.1 (resp. 7.8) by 4.778. The prevalence ratios (0.858 for men and 1.632 for women) were multiplied with the data from Table 3 (2nd column) to obtain HIV prevalences for men and women separately for all years between 1989 and 1998 (see Table 3, 3rd and 4th column).

### Calibration of the models

For the eight models, HIV transmission parameters were fitted to HIV prevalence data of Yaoundé from 1989 to 1998, described in the previous section. The parameter estimation procedure is described in Sect. 3 of the Supplementary Material.

Furthermore, we adapted the parameters for STI (HSV-2) transmission. In major epidemiological reviews of HSV-2^24–26^ the only data about Yaoundé that is mentioned is the data collected by Buvé et al.^27^ in the 4 Cities Study, in which the HSV-2 seroprevalence was 50% in females. Thus, we do not have information about the temporal trends of HSV-2 in Yaoundé. However, since the prevalence in Africa seems to have slightly declined between 2003 and 2012^22^ we assumed that the measured prevalence in Yaoundé, in 1997^27^, reflects an epidemic not far from its peak. Accordingly, we adapted the HSV-2 transmission parameters of the eight models so that the seroprevalence of this virus first increases and afterwards stabilizes at approximately 50% for females, corresponding to the HSV-2 prevalence in 1997 described by Buvé et al.^27^.

The estimated parameters and other key parameters for each of the eight models are described in the Supplementary Material, Sect. 4.

All models could be calibrated so that they fit the HIV prevalence data between 1989 and 1998, and the HSV-2 prevalence first increases and afterwards stabilizes at approximately 50% for females (see Supplementary Material, Sect. 5).

### Validation of the models against data not used for fitting

The eight models were validated against the HIV prevalence in 2004, which was not used for model calibration and has been reported to be 6.0% in males and 10.7% in females^28^. For each of the eight models, we checked how well these literature values could be predicted. For each model, 100 simulations were performed using the fitted parameters.

### Prediction of the impact of behavioural interventions

The goal of behavioural interventions is to reduce behaviour that increases the risk for acquiring HIV, and can be accomplished by increasing condom use or reducing the number of concurrent partners through individual counseling^29^. To investigate the impact of behavioural interventions on the HIV epidemic, the distribution of the number of partners was changed in all of the eight models to study the impact of behavioural change with respect to promiscuity. Only a single parameter was changed, we refer to this parameter as “behavioural change parameter”. Changing this parameter reduces the number of concurrent partnerships a person can have. For Simpact Cyan 1.0, the weight for the number of relationships a person already has (α_(numrel,man) = α_(numrel,woman) in formula (1)) was changed from 0 to − 0.05. For StepSyn 1.0, the proportion of male pending shorts links that are fulfilled by females (pending.short.links.fulfilled) was changed from 1 to 0.7. More details on the behavioural change parameter for Simpact Cyan 1.0 and StepSyn 1.0 are provided in Sect. 6 of the Supplementary Material.

Changing the behavioural change parameter in Simpact Cyan 1.0 and StepSyn 1.0, as described above and in Sect. 6 of the Supplementary Material has a similar effect on the distribution of the number of partners in both modelling frameworks:

- the mean number of partners is reduced by 6%;
- the 95th percentile is reduced with 1;
- the median and 75th percentile are unchanged.

To investigate what would have happened to the epidemic if promiscuity would have been lower from day 1 onwards, we implemented the change of the behavioural change parameter described above in 1980. Second, the intervention was applied in 1990 to study its impact when it would have been implemented at some point in time during the epidemic. The behavioural change in 1980 and 1990 is implemented in the same manner, but to draw attention to the distinction between a change at the start of the epidemic and an intervention during the course of the epidemic, we refer to the change in 1980 as “lower promiscuity” while referring to the change in 1990 as “behavioural intervention”.

For each model, 100 simulations with the fitted parameters, but changing the behavioural change parameter as described above, were run.

When studying the impact of interventions, only the parameter(s) related to the interventions (in this study only the behavioural change parameter) are changed. This means that we assume that apart from the intervention, no other influences are present. Therefore, the impact of behavioural interventions was also explored during the study period 1980–2005.

For each of the eight models, the impact of the intervention for females and males is calculated as the relative difference in cumulative HIV incidence at the end of the study period (2005) predicted by the model with and without intervention. As an additional measure, the difference in HIV prevalence in 2004 is reported in the Supplementary Material, Sect. 7.

## Results

### Quality of the model fits

All model fits show good quality, with no overfitting or underfitting (see Supplementary Fig. S12). A few small differences can be observed between the model fits, in particular for the last time point 1998. Most of the models, except the Si_IO_VL, St_RG and St_IO_RG models, slightly overestimate the HIV prevalence in 1998.

### Validation of the models against data not used for fitting

**Figure 1:**
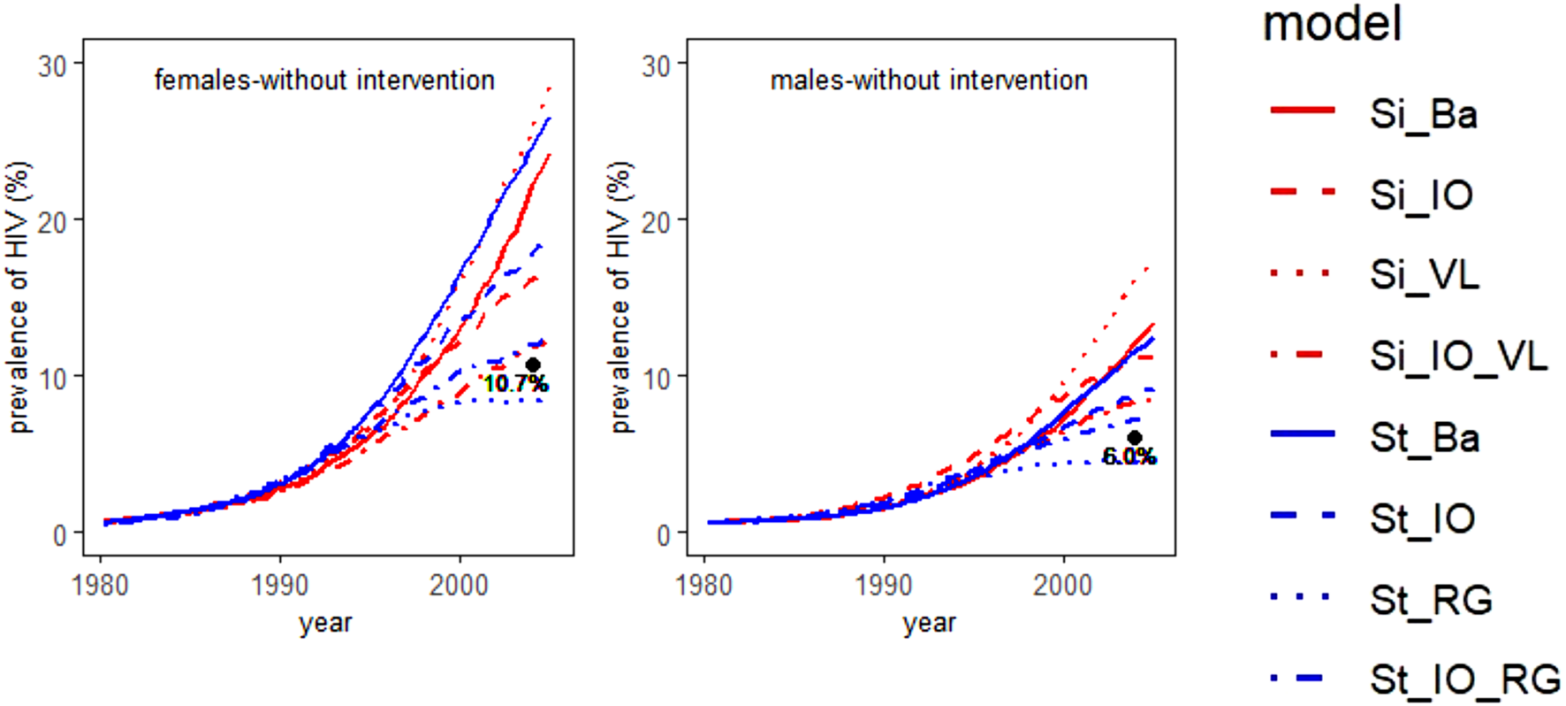
Prevalence curves for HIV from 1980 to 2005 in case no behavioural intervention is implemented. Median HIV prevalence (in %) of 100 simulations. The black dot represents the literature value from Ref.^28^. Left panel: females; right panel: males. Models: Si_Ba: Simpact 1.0 basic model; Si_IO: Simpact 1.0 model with inflow and outflow; Si_VL: Simpact 1.0 model with VL-dependent HIV transmission hazard; Si_IO_VL: Simpact 1.0 model with inflow, outflow, VL-dependent HIV transmission hazard; St_Ba: StepSyn 1.0 basic model; St_IO: Stepsyn 1.0 model with inflow and outflow; St_RG: StepSyn 1.0 model with STI life history explicitly modeled; St_IO_RG: StepSyn 1.0 model with inflow, outflow and STI life history explicitly modeled. Figures were generated using R software version 3.6.0. (R Core Team (2019). R: A language and environment for statistical computing. R Foundation for Statistical Computing, Vienna, Austria (URL https://www.R-project.org/)^14^.

The median HIV prevalence from 1980 to 2005 (in %), together with the literature value for the HIV prevalence in 2004^28^ is shown in Fig. 1. The relative squared error of the median predicted HIV prevalence in 2004 for females and males is presented in Table 4. All models simulating no population inflow and outflow, except the StepSyn 1.0 model with STI life history explicitly modelled (St_RG), largely overestimate the HIV prevalence in 2004 for both females and males. In contrast, the St_RG model underestimates the HIV prevalence in 2004 and the HIV prevalence stabilizes after 2000 and 2004 for females and males respectively. Implementing inflow and outflow considerably improves the prediction of the HIV prevalence for both females and males in 2004. For females, the Simpact 1.0 model including inflow, outflow and a VL-dependent HIV transmission hazard (Si_IO_VL) results in the prediction closest to the literature value (relative squared error of the median = 0.011). For males, the StepSyn 1.0 model with inflow, outflow and STI life history explicitly modeled (St_IO_RG) results in the best prediction of the value in the literature (relative squared error of the median = 0.034). In general, the StepSyn 1.0 models showed less variation between simulations in predicted HIV prevalence in 2004 than the Simpact 1.0 models (Supplementary Table S24). The largest variability among predictions (range of the simulation results without intervention) is observed for the Si_VL model, while the St_RG model has the lowest variability.

**Table 4:** Relative squared error of the median predicted HIV prevalence in 2004 for females and males (depicted in Fig. 1a,d).

Model	Females	Males
Si_Ba	1.142	1.035
Si_IO	0.267	0.681
Si_VL	2.048	2.868
Si_IO_VL	0.011	0.148
St_Ba	1.710	0.849
St_IO	0.445	0.211
St_RG	0.044	0.065
St_IO_RG	0.012	0.034

Models: Si_Ba: Simpact 1.0 basic model; Si_IO: Simpact 1.0 model with inflow and outflow; Si_VL: Simpact 1.0 model with VL-dependent HIV transmission hazard; Si_IO_VL: Simpact 1.0 model with inflow, outflow, VL-dependent HIV transmission hazard; St_Ba: StepSyn 1.0 basic model; St_IO: StepSyn 1.0 model with inflow and outflow; St_RG: StepSyn 1.0 model with STI life history explicitly modeled; St_IO_RG: StepSyn 1.0 model with inflow, outflow and STI life history explicitly modeled.

### Prediction of the impact of behavioural interventions

**Figure 2:**
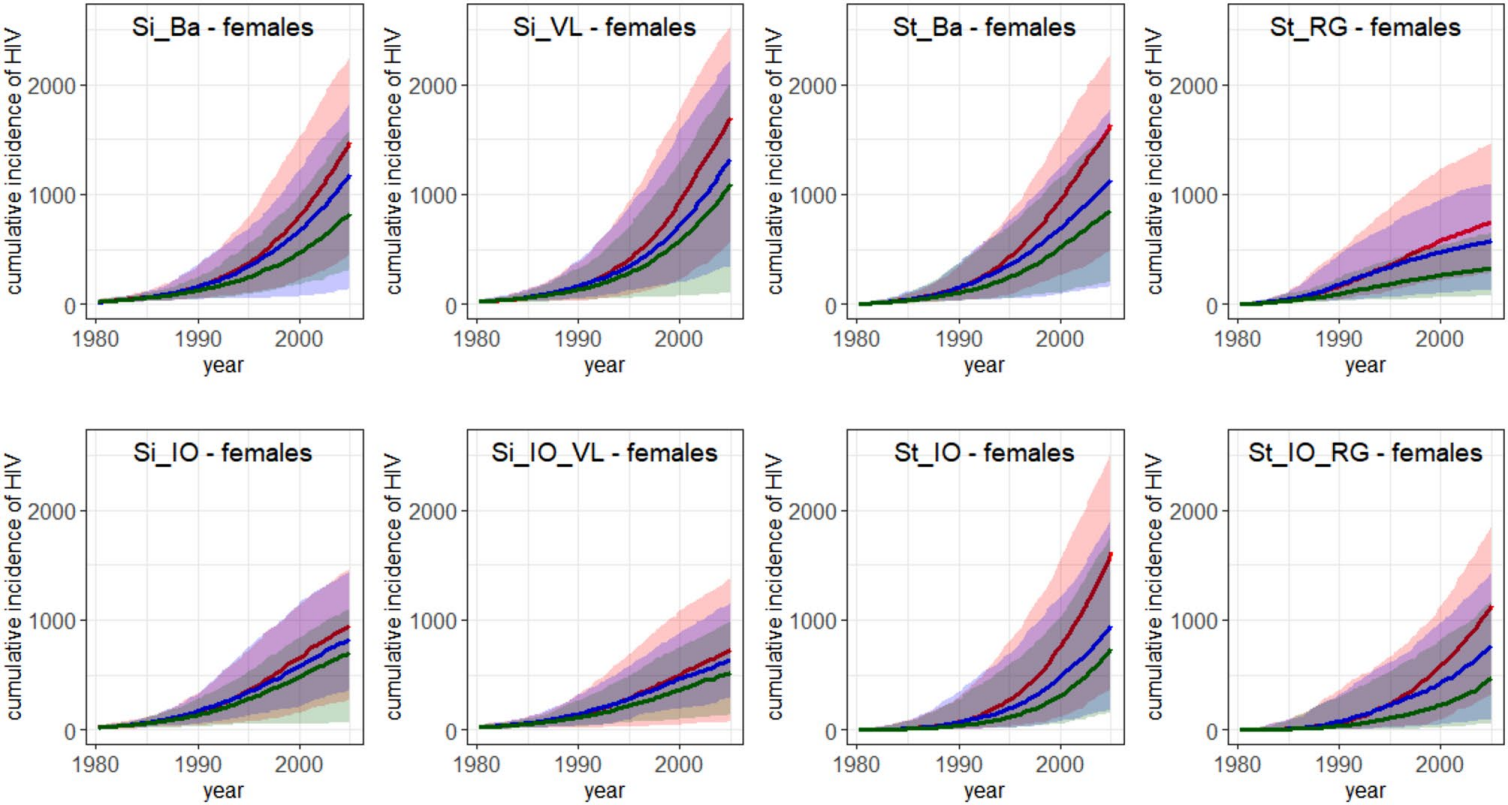
Cumulative incidence for HIV in females from 1980 to 2005. Median cumulative incidence of 100 simulations and the range ([minimum,maximum]) (shaded area). Red: no intervention implemented; blue: intervention implemented in 1990; green: lower promiscuity from 1980 onwards. Interventions were implemented as follows. For Simpact Cyan 1.0, the weight for the number of relationships a person already has (α_(numrel,man) = α_(numrel,woman) in formula (1)) was changed from 0 to − 0.05. For StepSyn 1.0, the proportion of male pending shorts links that are fulfilled by females (pending.short.links.fulfilled) was changed from 1 to 0.7. Model abbreviations as in Fig. 1. Figures were generated using R software version 3.6.0. (R Core Team (2019). R: A language and environment for statistical computing. R Foundation for Statistical Computing, Vienna, Austria (URL https://www.R-project.org/)^14^.

**Figure 3:**
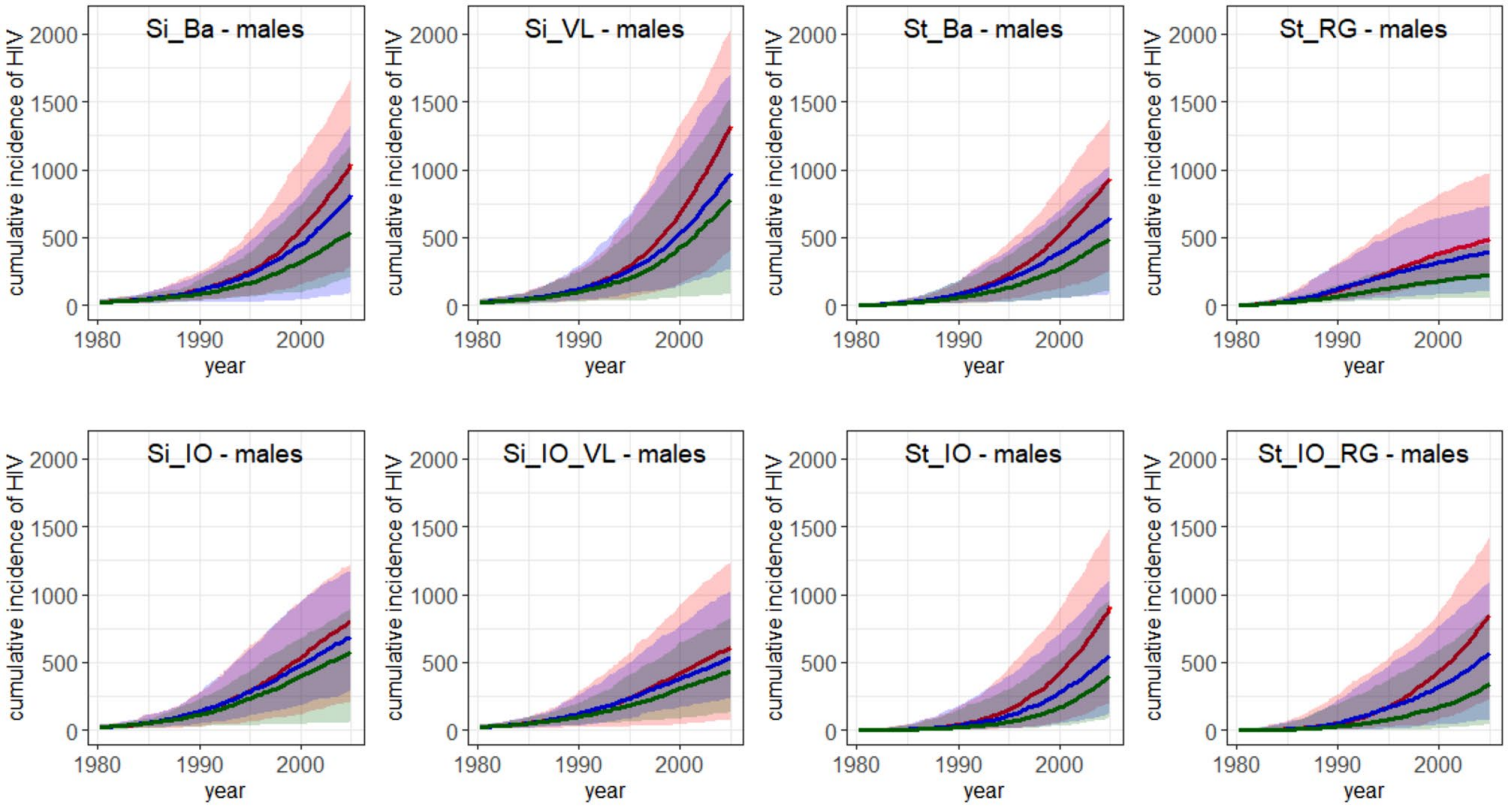
Cumulative incidence for HIV in males from 1980 to 2005. Median cumulative incidence of 100 simulations and the range ([minimum,maximum]) (shaded area). Red: no intervention implemented; blue: intervention implemented in 1990; green: lower promiscuity from 1980 onwards. Interventions were implemented as follows. For Simpact Cyan 1.0, the weight for the number of relationships a person already has (α_(numrel,man) = α_(numrel,woman) in formula (1)) was changed from 0 to − 0.05. For StepSyn 1.0, the proportion of male pending shorts links that are fulfilled by females (pending.short.links.fulfilled) was changed from 1 to 0.7. Model abbreviations as in Fig. 1. Figures were generated using R software version 3.6.0. (R Core Team (2019). R: A language and environment for statistical computing. R Foundation for Statistical Computing, Vienna, Austria (URL https://www.R-project.org/)^14^.

Figures 2 and 3 and Table 5 show that, in terms of difference in cumulative HIV incidence, a lower promiscuity from 1980 onwards has a similar effect on HIV relative to the Simpact Cyan 1.0 and StepSyn 1.0 basic models (Si_Ba and St_Ba). For the intervention in 1990, a larger effect is observed for the St_Ba model than for the Si_Ba model (see Table 5). In terms of HIV prevalence, both interventions have a similar effect for both basic models (see Supplementary Figs. S15, S16).

**Table 5:** Influence of interventions—relative difference in cumulative HIV incidence at the end of the study period (2005).

Model	Intervention in 1990		Lower promiscuity from 1980 onwards	
	Females (%)	Males (%)	Females (%)	Males (%)
Si_Ba	− 20	− 22	− 44	− 48
Si_IO	− 13	− 14	− 26	− 28
Si_VL	− 22	− 26	− 35	− 41
Si_IO_VL	− 12	− 12	− 29	− 28
St_Ba	− 31	− 30	− 48	− 48
St_IO	− 40	− 39	− 54	− 56
St_RG	− 23	− 19	− 56	− 54
St_IO_RG	− 32	− 32	− 58	− 60

Interventions were implemented as follows. For Simpact Cyan 1.0, the weight for the number of relationships a person already has (α_(numrel,man) = α_(numrel,woman) in formula (1)) was changed from 0 to − 0.05. For StepSyn 1.0, the proportion of male pending shorts links that are fulfilled by females (pending.short.links.fulfilled) was changed from 1 to 0.7. Median HIV incidence and range ([minimum,maximum]) of 100 simulations. Models abbreviations as in Fig. 1

Apart from reducing HIV, the interventions also reduce HSV-2 (see Supplementary Table S22), and the size of the reduction differs among the eight models and between interventions (intervention in 1990: reduction of the median cumulative HSV-2 incidence over the whole study period (number of new cases between 1980 and 2005): 1.9–16.3% (females) and 2.1–25.0% (males); lower promiscuity in 1980: reduction of 6.2–20.5% (females) and 6.7%–32.7% (males)).

Cumulative HIV incidence plots and relative differences in cumulative HIV incidence are provided in Figs. 2, 3 and Table 5. Median and range of cumulative HIV incidence in 2005 are provided in Supplementary Table S23. Median HIV prevalence in 2004 for females and males (in %) and range (the maximum minus the minimum) of 100 simulations in case of no intervention, a behavioural intervention in 1990 and lower promiscuity from 1980 onwards are shown in Supplementary Table S24.

Figures 2 and 3 show that for all models, the implementation of a behavioural intervention reduced the median cumulative HIV incidence and the range (see also Table 5). For the Simpact Cyan 1.0 models, the relative difference in cumulative HIV incidence was higher in the models without inflow and output, while in the StepSyn 1.0 models it was the opposite way around (see Table 5). Except for the models with STI history explicitly modeled, the intervention has a larger effect on the median HIV prevalence when implemented in models without inflow and outflow (see Supplementary Figs. S15, S16). After implementing a behavioural intervention in 1990, a reduced but still increasing HIV prevalence was observed for all models except the StepSyn 1.0 model with STI life history explicitly modeled (St_RG) which shows a decreased HIV prevalence after 1998 (Supplementary Fig. S17b,e). In case of lower promiscuity from 1980 onwards (Supplementary Fig. S17c,f), similar trends are observed but the reduction in HIV prevalence is considerably larger than when applying the intervention in 1990.

For the two models that resulted in the best predictions of the HIV prevalence in 2004 in case of no intervention (Si_IO_VL and St_IO_RG), the predicted effect of a behavioural intervention was larger for the St_IO_RG model than for the Si_IO_VL model (Table 5). While the Si_IO_VL model predicts a 12% reduction in median cumulative HIV incidence in 2005 for both females and males, in case a behavioural intervention was implemented in 1990, the St_IO_RG model predicts a 32% reduction for both females and males. In case of lower promiscuity from the start of the epidemic (1980), Si_IO_VL predicts a 29% and 28% reduction in median cumulative HIV incidence in 2005 for females and males respectively, while St_IO_RG model predicts a 58% and 60% reduction in females and males respectively. The larger effect of the interventions in the Si_IO_RG model can also be observed in the HIV prevalence in 2004 (Supplementary Table S24).

## Discussion

In this study, eight individual-based models, generated with the simulators Simpact Cyan 1.0 and StepSyn 1.0 were compared in terms of their prediction of the impact of behavioural interventions on the course of the HIV epidemic in the heterosexual population in Yaoundé, Cameroon during the period 1980–2005.

All models could be fitted equally well to the calibration targets in Table 3 (3rd and 4th column) (see Supplementary Fig. S12), which shows that similar HIV prevalence curves can be simulated using different model assumptions and transmission parameters.

After calibration of the models, they were first validated against data not used for fitting. For each of the two modelling frameworks, the model that implements population inflow and outflow together with a detailed description of HIV transmission (i.e. the Si_IO_VL and St_IO_RG models) shows the best prediction of the HIV prevalence for males and females in 2004 (Fig. 1).

In general, a model implementing inflow and outflow better predicts HIV prevalence at a time point not used for fitting than its counterpart without inflow and outflow (Fig. 1). Remarkably, while models with no inflow and outflow implementing a generic STI co-factor effect largely overestimated the HIV prevalence in 2004, the model implementing HSV-2 life history and its effect on HIV in detail, and no inflow and outflow (the St_RG model) predicted a lower HIV prevalence for both males and females than what was reported in the literature. This can be explained by the difference in parametrization between the generic and the detailed HSV-2 co-factor. The simplified parametrization in the models implementing a generic STI co-factor effect (2 parameters, see Supplementary Tables S10, S12, S14) compared to the models which implement HSV-2 stages and ulcerative recurrences (6 parameters, see Supplementary Table S16) results in higher estimates of the HIV prevalence. Absence of inflow and outflow strengthens this effect. This results in overestimates of the HIV prevalence for models with a generic STI co-factor effect, and underestimates for models with a detailed STI co-factor effect. Moreover, the effect of implementing inflow and outflow on the model predictions was smaller for the model implementing HSV-2 in detail than for the other models.

The Simpact Cyan 1.0 models showed a higher relative difference in cumulative HIV incidence in the models without inflow and outflow after implementing a behavioural intervention, while in the StepSyn 1.0 models it was the opposite way around (see Table 5). This can be explained by the differences in implementation of inflow and outflow between Simpact Cyan 1.0 and StepSyn 1.0. While Simpact Cyan 1.0 only implements birth and non-AIDS mortality, StepSyn 1.0 also implements immigration and emigration.

The predictions of the impact of behavioural interventions for the eight models are similar during the first five years after the intervention, but show large differences on the long term (see Figs. 2, 3, Supplementary Fig. S17b,c,e,f). Similar conclusions could be drawn by Eaton et al.^30^ when comparing mathematical models predicting the impact of antiretroviral therapy (ART).

All simplified Simpact Cyan 1.0 models (Si_Ba, Si_IO, Si_VL) overestimate the HIV prevalence in 2004 compared to the most detailed model (Si_IO_VL), and as a consequence these are the models with the highest HIV prevalence estimates. Furthermore, the simplified Simpact Cyan 1.0 models also have higher estimates of the proportion of serodiscordant couples (see Supplementary Table S25).

A behavioural intervention has a larger effect when implemented in a situation with higher HIV incidences and a higher proportion of serodiscordant couples. As a consequence, also larger effects of interventions on the cumulative HIV incidence are predicted in the simplified Simpact Cyan 1.0 models (Figs. 2, 3, Table 5). For the same reason, a smaller effect of interventions on the cumulative HIV incidence is observed for the StepSyn 1.0 models without inflow and outflow.

For StepSyn 1.0, the models that overestimate (St_Ba, St_IO) and underestimate (St_RG) the HIV prevalence in 2004, predict larger and smaller effects of behavioural interventions on HIV prevalence, respectively, compared to the most detailed model (St_IO_RG) (Table S24, Supplementary Figs. S15, S16, S18, S19). This can also be explained by the lower estimated proportion of serodiscordant couples in the St_RG model and the higher proportion of serodiscordant couples in the St_Ba and St_IO models (Supplementary Table S25).

For males, the baseline models (Si_Ba and St_Ba) are almost in perfect agreement (Fig. 1), but adding additional complexity to Si_Ba always leads to higher prevalence estimates whereas adding additional complexity to St_Ba always leads to lower prevalence estimates. The lower HIV prevalence estimates in StepSyn 1.0. can be explained by the inclusion of immigration and emigration (St_IO), the inclusion of a more detailed parametrization of the HSV-2 co-factor (St-RG) or both (St_IO_RG).

As the Si_IO_VL and St_IO_RG models showed the best predictions of the HIV prevalence in 2004 in females and males respectively, these models can be considered as the most reliable for predicting behavioural interventions.

Although the Si_IO_VL and St_IO_RG models predict the HIV prevalence in 2004 equally well, in particular in females (relative squared error 0.011 and 0.012 respectively), the two models behave differently when implementing the effect of a behavioural intervention. After an intervention in 1990, the median cumulative HIV incidences for females and males in 2004 were both reduced with 12% for the Si_IO_VL model (see Table 5), while the St_IO_RG model predicts a reduction of 32% for both females and males. In case of lower promiscuity from 1980 onwards, a reduction in median cumulative HIV incidence of 29% for females and 28% for males were predicted by the Si_IO_VL model. For the St_IO_RG model, the corresponding values were 58% and 60%. The larger effect of the interventions in the Si_IO_RG model could also be observed in the differences in cumulative HIV prevalence (Supplementary Table S24). The difference in the prediction of the effect of the behavioural intervention can be explained by several factors. First, the lower cumulative HIV incidences in the St_IO_RG model compared to the Si_IO_VL model can be explained by the more detailed parametrization of the HSV-2 co-factor effect (see above) and the inclusion of immigration and emigration in the St_IO_RG model (see Table 2). Second, lower promiscuity is known to have a positive effect on viral load^31^ and also lowers the risk of acquiring an STI/HSV-2. The differences between the outputs of the simulations could be explained by differences in the sizes of the effect of lower promiscuity on viral load and STI co-factors. More data has to become available, in particular more detailed information on the relationship between sexual risk behaviour, HIV viral load and STI co-factors, to further explore which of these two models provides the most reliable predictions of behavioural interventions. As studies also suggest that HSV-2 influences HIV viral load^32^, it is recommended to combine the predictions of the St_IO_RG and Si_IO_VL models using ensemble modeling. When comparing the estimates of the proportion of serodiscordant couples for the Si_IO_VL and St_IO_RG models with the literature value of 14.4% in 2004, reported in Ref.^28^, we notice that the estimated value for the Si_IO_VL model (15.0%) is the closest to the reported value (Supplementary Table S25). For the HIV prevalence in males in 2004, the estimated value for the St_IO_RG model is the closest to the reported value (see Fig. 1).

Seven of the eight models predicted that the HIV prevalence would still increase during the period 1980–2005, although at a lower rate, after applying a behavioural intervention (see Supplementary Fig. S17b,c,e,f). Only the St_RG model predicted that the HIV prevalence will decrease after reaching a peak in 1997. As mentioned earlier, including detailed HSV-2 stages underestimates HIV prevalences in absence of inflow and outflow, and predicts a stabilizing HIV prevalence in case of no intervention (Fig. 1). As a consequence, a decreasing HIV prevalence was predicted when a behavioural intervention was implemented. The difference between the predictions can also be explained by the lower estimated proportion of serodiscordant couples in the St_RG model compared to the other StepSyn 1.0 models (Supplementary Table S25).

This study shows that differences in model assumptions and model complexity can considerably influence their predictions of the impact of behavioural interventions. Hontelez et al.^33^ reported similar conclusions after stepwise inclusion of model complexity in a model for predicting the impact of a universal test and treat (UTT) intervention and concluded that sufficient detail is necessary to make accurate predictions.

Apart from differences in the size of the impact of a behavioural intervention on the HIV epidemic, we also detected differences in qualitative behaviour between simulations generated with different models.

Our study has several limitations. First, to be able to compare the models generated with the two modeling frameworks, our study was restricted to a population that can be handled by both StepSyn 1.0 and Simpact 1.0. Therefore, men having sex with men (MSM) and commercial sex workers (CSW) were not included in this study. To the best of our knowledge, no data on HIV in MSM in Yaoundé are available for the study period 1980–2005. A study from 2014 estimated that the proportion of males being MSM is 1.38% (95% confidence interval (CI) 0.51–2.25%^34^. In 2011, the reported HIV prevalence in MSM in Yaoundé was 44.4%^35^. Because of the low proportion of MSM in the male population, we expect that including HIV transmission in MSM in mathematical models for the whole sexually active population would only lead to a small increase in the HIV prevalence in males, and therefore recommend to study MSM as a separate population in HIV modeling studies. The proportion of females in Yaoundé being CSW is estimated 1.88% (95% CI 1.15–2.61)^34^, and the reported HIV prevalence in CSW was 34.8% in 1997^36^. Therefore, including CSWs will also results in higher HIV prevalence estimates.

Second, the intervention had to be chosen such that it could be simulated with both Simpact Cyan 1.0 and StepSyn 1.0 frameworks. As treatment of HIV with antiretroviral therapy (ART) is not implemented in StepSyn 1.0, male circumcision is not available in Simpact Cyan 1.0 and none of the two modeling frameworks has an option to simulate condom use, we decided to simulate an intervention reducing the number of partners (by e.g. individual counseling). Because ART for HIV was not implemented in both frameworks, the study period had to be restricted to 1980–2005. As an increase in ART coverage from 0% in 2003 to 22% in 2014 has been reported^37^, the model assumption of no ART in our model will not be valid for more recent time periods. The interventions not modelled in this study (ART, condom use, male circumcision) are all expected to reduce HIV prevalence.

Third, mother-to-child transmission (MTCT) was not implemented, which means that all individuals in the simulation that enter the population through birth are assumed to be HIV negative. Implementation of MTCT will be a direction for future research.

Fourth, there is a need for accurate estimates of the lifetime number of partners for evaluating the dynamics of partnership formation and dissolution in mathematical models. These estimates currently rely on self-reported values. For the heterosexual population in Yaoundé, these data are reported by Ferry et al.^19^ (median and interquartile range (IQR)) and the 2004 Demographic Health Survey (DHS)^28^ (mean). Reported values were based on a questionnaire from UNAIDS^38^, which determines the lifetime number of partners as the number of partners up to the date participants filled in the questionnaire. When comparing these literature values with estimates from the mathematical models (see Supplementary Material, Sect. 9 for more detail), we observe that the large discrepancy in lifetime number of partners between males and females (median of 10 and 3 respectively) reported in the literature is not reflected by the mathematical models in this study (Supplementary Table S26). This does not necessary invalidate the models, as reliability of self-reported sexual behaviour data, in particular the discrepancy between females and males, has been subject to debate^39^. Furthermore, which model value is closest to the literature value depends on gender and the literature source (Supplementary Table S26). For the median and IQR from Ferry et al.^19^, the values of the St_IO and St_IO_RG models are closest to the literature values. For males, the numbers reported by the St_Ba and St_RG models are closest to the those in Ferry et al. (but for females they are the furthest from the literature value). For the mean reported in the 2004 DHS^28^, the value from the St_IO_RG and Si_IO model are closest to the literature average for females and males respectively.

Because of the limitations above, the models described in this study are only partial descriptions of reality, a property of all mathematical models in epidemiology. Although the models are highly simplified, they can still provide meaningful information^40^ and it still makes sense to compare the models in the current phase of their development. Comparing different models also stimulates to extend them, but therefore it is necessary to have sufficient data available. Because of the large uncertainty in several epidemiological parameters, it is also important to have different models with different assumptions, and to combine these models using ensemble methods^41,42^. Several studies have shown that combining models using ensemble approaches considerably improve prediction accuracy compared to predictions from individual models. Examples are ensembles of transmission models for the burden of dengue^43^, ensembles of epidemiological models to study the impact of vaccination strategies^44,45^, the RAPIDD ebola forecasting challenge which combined 8 independent modelling approaches^41^ and an ensemble model combining 20 mathematical models for seasonal influenza^42^.

Finally, only limited data were available that could be used for model validation. We showed that even when there is an agreement between two models in their prediction of a future time point not used for fitting, they can have different outputs when simulating the impact of interventions. Without more HIV prevalence data for validation, it is not possible to determine which of these two models is the most reliable. In case more HIV prevalence data would have been available for the period 1999–2005 than only the HIV prevalence in 2004, validation could have been performed on multiple data points not used for calibration. This would have enabled to better determine which model has the best prediction of the epidemic for the period 1999–2005. These findings highlight the importance of making more data available for both the calibration and validation of epidemiological models that aim to inform decisions made by policy-makers.

## Supplementary Information

Supplementary Information.

## Data Availability

The data used to calibrate the models in this study, together with references supporting these data, are available within the Supplementary Material, Sect. 2. The R-scripts that have been used for fitting HIV transmission parameters and the data sets of simulation results (HIV prevalence and cumulative HIV incidence) are available from GitHub: https://github.com/dmhendrickx/Scripts_comparison_Simpact_StepSyn. Simpact Cyan is freely available from http://www.simpact.org/. StepSyn is not yet freely available but will become so upon publication of a manuscript that describes the modelling framework into more detail (manuscript in preparation). In the meanwhile, the StepSyn code is available from KU Leuven (contact: João Dinis Sousa, e-mail: joao.sousa@kuleuven.be) on reasonable request.
